# Anti-tumor activity of stem cell-derived extracellular vesicles

**DOI:** 10.18632/oncotarget.26759

**Published:** 2019-03-08

**Authors:** Alessia Brossa, Valentina Fonsato, Benedetta Bussolati

**Affiliations:** Department of Molecular Biotechnology and Health Sciences, University of Torino, Torino, Italy

**Keywords:** stem cells, tumor, exosomes, microRNA, angiogenesis

Extracellular vesicles (EVs) have gathered a substantial amount of interest over the past years, as an intercellular communication system for biologically active molecules, such as mRNAs, miRNAs, lipids and proteins [1]. The ability of stem cell-EVs to reprogram recipient cells has been demonstrated in different tissues and pathologies. In oncology, several groups approached the possible use of stem cell-derived EVs to inhibit tumor growth and progression, with conflicting results.

Recently, Lopatina *et al.* demonstrated that EVs derived from a resident population of human liver stem cells with mesenchymal characteristics exert anti-angiogenic effect on tumor-derived endothelial cells (TEC) from renal carcinomas. This effect was confirmed *in vivo*, when systemically injected EVs affected human TEC-derived vessels connected with the mouse vasculature [2]. Of relevance, liver stem cell-EVs showed a selective effect on TECs, as they were ineffective on normal endothelial cells. This could be explained by the activity of EVs on altered gene expression profile of tumor, and not normal, endothelium. Indeed, it is well known that TECs display an altered phenotype, and that they might derive from modified endothelial cells recruited from tumor adjacent vessels as well as from an intra-tumor vasculogenesis [3]. Concerning this possibility, renal TECs might originate from the endothelial differentiation of cancer stem cells (CSCs), a well-recognized sub-population of tumor cells able to sustain the initiation, the maintenance and the recurrence of solid tumors [3]. Of interest, it has been recently demonstrated that liver stem cell-EVs also induce apoptosis of renal CSCs, alone or in combination with drugs commonly used in clinical practice, highlighting a double, although different, effect of liver stem cell-EVs on both tumor endothelial and cancer stem cells [2, 4].

Tumor angiogenesis might be differently modulated by stem cell-derived EVs of different origin. For instance, mesenchymal stem cell (MSC)-EVs were shown to promote tumor vascularisation in gastric and colon cancer models [5]. At variance, Lee *et al.* described the *in vitro* and *in vivo* inhibition of angiogenesis driven by MSC-EVs through vascular endothelial growth factor down-regulation in breast cancer [6]. In the paper of Lopatina *et al.*, MSC-EVs, at variance of liver stem cell-EVs, failed to inhibit tumor angiogenesis, [2] and similar lack of activity was also reported in a recent study on murine lymphomas [7]. This variable activity of stem cell-EVs may possibly depend on the type of stem cell originating EVs as well as on their target. In this context, EVs from MSCs rather appear to display a general pro-angiogenic activity, of value for tissue regeneration. At variance, cardiac stem cell-derived EVs were able to reduce tumor vascularization in parallel with tumor proliferation, *in vitro* and *in vivo* [7]. Other reported sources of EVs with anti-tumor activity were human embryonic stem cell [8] and placental stem cells [9].

**Figure 1:**
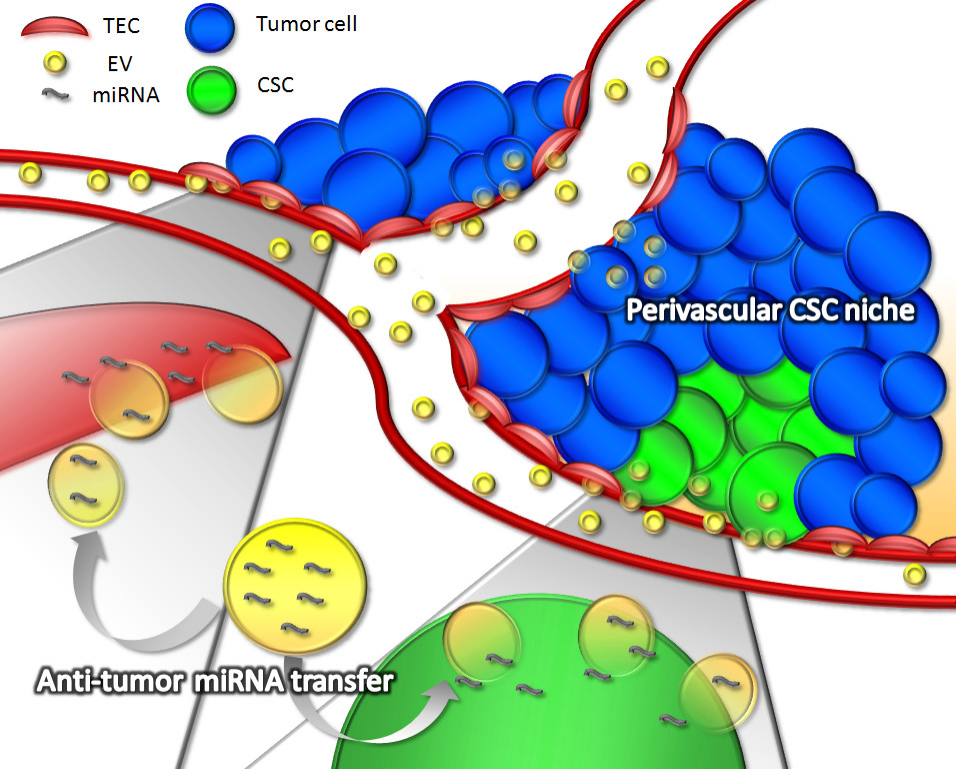
Anti-tumor effect of stem cell-derived Evs Stem cell-derived EVs could act in different steps of tumor progression, by targeting tumor endothelial cells, cancer stem cells and differentiated tumor cells. The direct transfer of specific anti-angiogenic miRNAs, (such as miR-181b, miR-320c, miR-874, miR-16 and miR-146), by stem cell-derived EVs to endothelial cells may result in reduced tumor vascularization. Due to the aberrant vascularization of tumors, and to the presence of fenestrations in the endothelium, EVs could efficiently reach both tumor epithelial cells and cancer stem cells in their perivascular niche, where the transfer of other anti-tumor miRNAs, such as miR-451, miR-31, and miR-223, may affect specific pathways responsible for tumor growth and survival.

The described effect of stem cell-EVs on tumor cells has been mainly ascribed to the transfer of specific miRNAs able to reprogram their gene expression profile (Figure 1). In particular, among the anti-angiogenic miRNAs identified in liver stem cell-EVs, miR-181b, miR-320c and miR-874 were described as responsible of the down-regulation of tumor angiogenic factors such as FGF1 and PLAU [2]. In addition, miR-16, enriched in MSC-EVs, was also reported to contribute to vascular endothelial growth factor down-regulation in breast cancer cells [6] and miR-146, highly enriched in cardiac stem cell-EVs, was implicated in their anti-angiogenic and anti-tumor effects [7]. Moreover, liver stem cell-EVs were shown to transfer other anti-tumor miRNAs, such as miR- 451, miR-31, and miR-223 to hepatoma cells, inducing a down-regulation of their targets (MDR1, RAB14, MIF and E2F-2) [10]. As result, intracellular pathways relevant for tumor growth and survival, such as Akt/mTOR/PTEN, were down-regulated by EV treatment [4]. In addition, a synergistic activity of stem cell-EVs was observed with tyrosine kinase inhibitors, enhancing the blockade of tumor intracellular pathways in CSCs [4].

Altogether, these data provide a rationale for the use of stem cell-derived EVs, from a number of different stem cell sources, for the treatment of solid tumors. Of interest, stem cell-derived EVs appear to act on multiple targets such as tumor endothelial cells, tumor stem cells as well as on differentiated cells (Figure 1). Indeed, stem cell-EVs may display a unique anti-tumor effect thanks to a variety of molecular species within their cargo and to an orchestrated activity on multiple pathways.
